# 4D-DIA Proteomic Analysis of IPEC-J2 Cells Infected with Porcine Group A Rotavirus G9P[23] Strain

**DOI:** 10.3390/vetsci12100946

**Published:** 2025-09-30

**Authors:** Zhendong Zhang, Yubo Li, Xingyu Zhou, Duo Li, Muyao Li, Xueyang Wang, Qinghai Ren, Xiaowen Li

**Affiliations:** 1 Jiangsu Co-Innovation Center for Prevention and Control of Important Animal Infectious Diseases and Zoonoses, College of Veterinary Medicine, Yangzhou University, Yangzhou 225009, China; someonelyb@163.com (Y.L.); li15831216053@163.com (D.L.);; 2 School of Biotechnology, Jiangsu University of Science and Technology, Zhenjiang 212018, China; xueyangwang@just.edu.cn; 3 College of Agriculture and Biology, Liaocheng University, Liaocheng 252000, China; renqinghai@lcu.edu.cn

**Keywords:** porcine group A rotavirus, 4D-DIA proteomic analysis, IPEC-J2 cells, host-virus interaction, inflammatory response

## Abstract

Porcine rotavirus (PoRV) is one of the most devastating enteric pathogens causing gastroenteritis in pigs, particularly the sudden occurrence in recent years in China. To elucidate host–pathogen interactions and molecular mechanisms underlying PoRV pathogenesis, four-dimensional (4D) data-independent acquisition (DIA) proteomic (4D-DIA) analysis was performed to comprehensively quantify the differentially abundant proteins (DAPs) in PoRV-infected IPEC-J2 cells. A total of 8725 cellular proteins were identified with 279 more abundant and 356 down abundant proteins. A Western blot showed that the abundance of SA100A8, DAPK2, and FTL were in accordance with the acquired proteomic data using 4D-DIA analysis. Bioinformatics analyses of GO and KEGG demonstrated that various DAPs are involved in crucial biological processes and signaling pathways, such as immune response, signal transduction, metabolic pathways, autophagy, endoplasmic reticulum (ER) stress, and mitochondrial dysfunction. Notably, inflammatory features of host response upon PoRV infection were highlighted, with RT-qPCR confirming the significant upregulation of IL-1α, IL-6, IL-8, TNF-α, STAT1, and IRF9 transcript levels during infection. Altogether, our preliminary findings advance our understanding of PoRV pathogenesis and may shed light on identifying potential targets for the prevention and control of PoRV-associated gastroenteritis.

## Simple Summary

Porcine rotavirus (PoRV) is a significant pathogen causing gastroenteritis in pigs and has recently emerged as an epidemic in China. In this study, 4D-DIA proteomic analysis was performed on PoRV-infected IPEC-J2 cells, identifying 8725 cellular proteins, including 279 more abundant and 356 under-detected proteins. Western blot validation confirmed consistent expression patterns of S100A8, DAPK2, and FTL with the proteomic data. Bioinformatics analysis revealed that these DAPs were involved in critical biological processes such as immune response, signal transduction, and metabolic pathways. RT-qPCR analysis demonstrated pronounced inflammatory responses during infection. These findings provide critical insights into pathogenic mechanisms and host defense strategies underlying PoRV infection.

## 1. Introduction

Porcine rotavirus (PoRV), first identified and isolated in the 1970s, is a non-enveloped virus with a genome composed of eleven segments of double-stranded RNA (dsRNA), which is classified in the *Rotavirus* genus within the *Sedoreoviridae* family by the International Committee on Taxonomy of Viruses (ICTV) [1,2]. Distinguished on the genetic and antigenic properties of the VP6 structural protein, rotaviruses are categorized into A-J groups. While groups A, B, C, H, and E rotavirus have been detected in pigs, diarrheal diseases associated with group A PoRV (PoRVA) infection were historically considered to be the most prevalent and pathogenic within global swine herds [2,3,4]. Clinically, PoRVA-infected pigs often present with watery diarrhea, dehydration, weight losses, and the death of piglets, with mortality rates elevated in neonatal or weaned piglets, particularly in some farms with suboptimal management practices [5,6]. Recent epidemiological surveys highlight the growing prevalence in China, Qiao et al. revealed that 86.52% of pig farms were positive for PoRVA, with an overall detection rate of 51.15% in clinical samples in 2022 [7]. A subsequent 2024 study analyzing 2422 diarrheal specimens across 26 provinces identified an average PoRVA positivity rate of 42% [8]. Obviously, these findings underscore a marked increase in PoRVA incidence over the past years, leading to considerable economic losses to the swine industry, but there are still no effective measures to prevent and control the PoRV-associated diseases. Of additional public health concern, pigs harboring rotavirus have been recognized as the important reservoirs for interspecies transmission. Genetic reassortment between porcine and human rotaviruses poses a potentially severe threat for human health [9,10].

In our previous study, the PoRV strain AHBZ2304 (G9P[23]) was exhibited to be highly pathogenic for newborn piglets, with gut microbiota dysbiosis and inflammatory cytokine overexpression identified as key contributors to intestinal pathogenesis [11]. Although extensive research has elucidated the rotavirus life cycle, limited studies were focused on the pathogenic mechanisms and immunomodulation of porcine rotavirus. Host transcriptomic and proteomic alterations commonly occur during viral infection, offering critical insights into these processes. For instance, Raque et al. conducted comparative transcriptome responses of G5P[7] and G9P[13] PoRVA strains in porcine ileal enteroids (PIEs), revealing immune response activation and cholesterol metabolism modulation as pivotal host responses [12]. Similarly, Raev et al. revealed antiviral factors that may be the mechanisms responsible for the unique rotavirus C characteristics through transcriptome analysis [13]. Compared to transcriptome, proteomics is likely to be more effective for the comprehensive analysis of host cellular responses to viral infections. Lv et al. performed proteomic analysis upon porcine endemic diarrhea virus (PEDV) infection and demonstrated that differentially abundant proteins (DAPs) were involved in immune response regulation, signal transduction, lipid transport, and metabolism processes as well as cell apoptosis pathways [14]. Further studies by Shen et al. and Huang et al. characterized the proteomic alterations of exosome and membrane during PEDV infection, respectively, uncovering roles for exosome-mediated signaling and PARD3 in viral pathogenesis [15,16]. Guo et al. expanded the work by comparing proteomic signatures of virulent and CV777 vaccine strain-like PEDV strains [17]. However, proteomic investigations of PoRV remain scarce.

As an enteropathogenic virus, PoRV targets the small intestine as the primary infected organ and mainly replicates in the porcine small intestinal epithelial cells (IPEC) [18]. The IPEC-J2 cell line, originally derived from the jejunum of newborn piglets, retains the physiological and morphological characteristics of primary IPEC and serves as a well-established in vitro model for studying porcine enteric virus–host interactions. Four-dimensional (4D) data-independent acquisition (DIA) proteomics, as an emerging technology tool, provides a comprehensive, high-throughput, and high-sensitivity platform for biopharmaceutical research and has been widely applied [19]. In the present study, 4D DIA-based quantitative proteomics was first employed to systematically quantify global proteomic changes in PoRVA AHBZ2304 (G9P[23])-infected IPEC-J2 cells, hoping to elucidate porcine rotavirus–host interaction networks and provide some clues for studying the pathogenic mechanisms and novel therapeutic methods in the future.

## 2. Materials and Methods

### 2.1. Virus, Cells, Antibodies, and Chemicals

The PoRVA AHBZ2304 strain (GenBank access number: PP683069–PP683071) was previously isolated from diarrheic samples in our research [5]. IPEC-J2 cells were cultured in Dulbecco’s modified Eagle medium F-12 (DMEM/F12) (Gibco, Shanghai, China) supplemented with 10% fetal bovine serum (NULEN BIOTECH, Shanghai, China) and maintained in a humidified 5% CO_2_ incubator at 37 °C. The primary antibodies used in the study were specific for SA100A8, DAPK2, FTL (Abclonal, Wuhan, China), Tubulin (NULEN BIOTECH, Shanghai, China), and VP6 (prepared in our lab). The HRP-labeled secondary antibodies against mice were purchased from Abclonal (Wuhan, China) and Alexa fluor 555-conjugated anti-mouse IgG antibody from Cell Signaling Technology (Danvers, MA, USA).

### 2.2. Virus Inoculation

IPEC-J2 cells reaching 80% confluence were rinsed twice with sterile phosphate-buffered saline (PBS) and then mock infected or infected with PoRVA AHBZ2304 strain at a multiplicity of infection (MOI) of 1. After adsorption for 1.5 h at 37 °C, the cells were rinsed once with sterile PBS, then serum-free DMEM/F12 medium containing 4 μg/mL of trypsin (Sigma-Aldrich, Bayswater, VIC, Australia) was added. The cells were cultured at 37 °C for 48 h, and infection was confirmed through cytopathic effect (CPE) observation, immunofluorescence assay (IFA), and Western blot analysis, as described in our prior work [5].

### 2.3. Protein Extraction and Preparation

Three independent experiments were performed to serve as biological replicates. At 24 h post-infection (HPI), PoRV (AHBZ2304)-infected and mock-infected IPEC-J2 cells (grown in 10 cm cell culture dishes) were rinsed twice with pre-cooling PBS and harvested into 2 mL EP tubes using trypsin digestion, following 500 uL of lysis buffer (1% sodium deoxycholate (SDS), 8 M urea) supplemented with phenylmethanesulfonyl fluoride (PMSF) protease inhibitor (Beyotime Biotechnology, Shanghai, China). The mixture was vortexed to mix well and incubated at 4 °C for 30 min. Cellular debris was removed by centrifugation at 12,000 rpm for 20 min at 4 °C, then the protein concentration was determined using Bicinchoninic acid (BCA) method by BCA Protein Assay Kit (Beyotime Biotechnology, Shanghai, China). Protein sample preparation was conducted using a commercial iST Sample Preparation kit (PreOmics, Planegg, Germany) according to the manufacturer’s protocols, mainly containing the process of protein denaturation, reduction, and alkylation, as well as the tryptic digestion and peptide purification.

### 2.4. LC-MS Detection and Data Analysis

An UltiMate 3000 liquid chromatography system (Thermo Fisher Scientific, Waltham, MA, USA) was coupled with a timsTOF Pro2 Mass Spectrometer (Bruker Daltonics). Samples were reconstituted in 0.1% formic acid (FA), and 200 ng peptide was separated using an AUR3-15075C18 column (15 cm length, 75 μm i.d, 1.7 μm particle size, and 120 Å pore size, IonOpticks, Collingwood, VIC, Australia), with a 60 min gradient starting at 4% buffer B (80% acetonitrile (can) containing 0.1% FA), followed by a stepwise increase to 28% over 25 min, 44% over 10 min, and 90% over 10 min (maintained for 7 min), then equilibrated at 4% for 8 min. The column flow rate was maintained at 400 nL/min, with a column temperature of 50 °C. DIA was performed in the diaPASEF mode, and data was processed and analyzed by Spectronaut 18 (Biognosys AG, Schlieren, Switzerland) with default settings. A total of 22 × 40 Th precursor isolation windows were set, ranging from *m*/*z* 349 to 1229. To match the MS1 cycle time, we set the repetitions to variable steps (2–5) in the 13-scan diaPASEF protocol. During PASEF MSMS scanning, the collision energy was ramped linearly as a function of the mobility from 59 eV at 1/K_0_ = 1.6 Vs/cm^2^ to 20 eV at 1/K_0_ = 0.6 Vs/cm^2^.

For data analysis, specific trypsin was set as the digestion enzyme; carbamidomethyl on cysteine was specified as the fixed modification, oxidation on methionine was specified as the variable modifications, and retention time prediction type was set to dynamic iRT. Data extraction was determined by Spectronaut based on the extensive mass calibration. Spectronaut will determine the ideal extraction window dynamically depending on iRT calibration and gradient stability. Q value (false discovery rate, FDR) cutoff was set to 1% at both the precursor and protein levels. Decoy generation was set to mutated, which is similar to scrambled but will only apply a random number of AA position swamps (min = 2, max = length/2). Local normalization was adopted as the normalization strategy. Peptides passing the 1% Q-value cutoff were used to quantify major groups via the MaxLFQ method.

### 2.5. Bioinformatics Analysis

To identify differentially abundant proteins (DAPs) between the PoRV-infected group and the mock-infected group, fold change (FC) was calculated as the mean ratio of each protein’s quantitative values across all biological replicates. The *t*-test was applied to compare protein quantitative values between the two groups, and the corresponding *p*-value was computed for statistical significance testing. DAPs were filtered with the criteria of fold change > 1.2 or <0.83 and *p*-value < 0.05. Bioinformatics analysis of the identified DAPs was performed using Omicsmart (http://www.omicsmart.com), a real-time interactive online platform for data analysis, including Gene Ontology (GO) analysis and KEGG pathway analysis.

### 2.6. RNA Extraction, Real-Time PCR, and Western Blot Analysis

Total RNA was extracted from PoRV-infected IPEC-J2 cells and the mock-infected group using TRIzol reagent (Invitrogen, Carlsbad, CA, USA). Subsequently, 1 μg of total RNA was reverse transcribed using HiScript IV 1st Strand cDNA Synthesis Kit (+gDNA wiper) (Vazyme, Nanjing, China). Quantitative real-time PCR (RT-qPCR) was performed using ChamQ SYBR qPCR Master Mix (Vazyme, Nangjing, China) and specific primers (Supplementary Table S1). The amplification reaction was programmed as follows: initial denaturation at 95 °C for 30 s, followed by 40 cycles of 95 °C for 5 s and 60 °C for 31 s. Three biological replicates were analyzed, each with three technical replicates. GAPDH gene served as the internal reference, and a relative quantitative method (2^−ΔΔCt^) was calculated to assess differences.

To further validate the identified DAPs, mock and PoRV-infected cells were collected for Western blotting to analyze S100A8, DAPK2, and FTL protein abundance levels using Tubulin as internal control. Briefly, equivalent amounts of cell lysates were mixed with 6× sample loading buffer, boiled for 10 min, and then separated on 12% SDS–PAGE gels. The proteins were electro-transferred to PVDF membranes (Millipore, Burlington, MA, USA), following a block with 5% skim milk with TBST (Tris-HCl-buffered saline solution + Tween) and then incubated with primary antibodies (above) overnight at 4 °C. The membranes were washed with TBST three times and then incubated with HRP-conjugated secondary antibody for 1 h at 37 °C. The signals were obtained using the clarity-enhanced chemiluminescence (ECL) reagent (Solarbio, Beijing, China).

### 2.7. Statistical Analysis

All data were processed and analyzed using GraphPad Prism 8.0 software (GraphPad, San Diego, CA, USA). The values were presented as means ± standard deviation (SD), and Student’s *t*-test was used to analyze the difference between the values of the two groups. *p*-values less than 0.05 were considered statistically significant: *, *p* < 0.05; **, *p* < 0.01; ***, *p* < 0.001; and ****, *p* < 0.0001.

## 3. Results

### 3.1. Replication of PoRV G9P[23] in IPEC-J2 Cells

**Figure 1:**
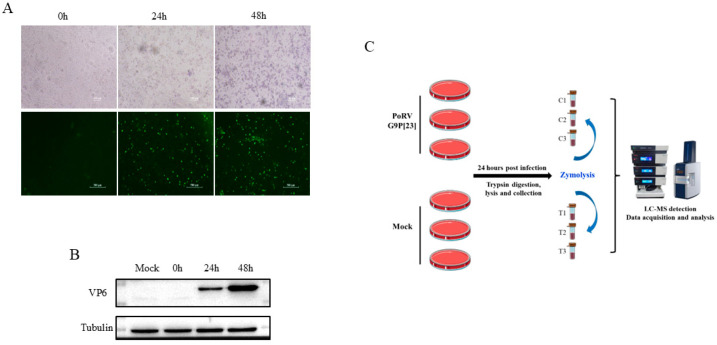
The infection of PoRVA strain AHBZ2304 in IPEC-J2 cells and workflow of proteomic analysis. (**A**) Morphological changes and immunofluorescence staining of AHBZ2304 or mock-infected IPEC-J2 cells at 24 HPI and 48 HPI. (**B**) Western blot analysis of AHBZ2304 VP6 expression levels of AHBZ2304-infected IPEC-J2 cells at 24 HPI and 48 HPI. (**C**) Experimental design and workflow of proteomics using 4D-DIA approach.

The PoRVA strain AHBZ2304 (G9P[23]), originally isolated from MA104 cells, was propagated in IPEC-J2 cells for three serial passages to confirm productive replication. Viral titer was determined using TCID_50_ assay. The IPEC-J2 cells were then infected with AHBZ2304 at a multiplicity of infection (MOI) of 1, and the VP6 protein expression was analyzed using an immunofluorescent assay (IFA) and Western blot. As shown in Figure 1A,B, the mock-infected cells exhibited no detectable cytopathic effects (CPEs) or VP6-specific signals, whereas infected cells displayed pronounced CPEs, distinct VP6 immunofluorescence, and robust VP6 band intensity on Western blots, in agreement with the results in MA104 cells [5]. Notably, the CPEs, fluorescence, and protein gray increased progressively over time, confirming active viral replication in IPEC-J2 cells. Previous studies had demonstrated that most RVs complete their first replication cycle at six HPI [20]. Thus, IPEC-J2 cells infected with one MOI PoRV for 24 h were selected to capture downstream proteomic alterations following the early stage of infection. To ensure the credibility of the acquired proteomic data, three biological replicates of infected and mock-infected cells were processed for 4D DIA-based quantitative proteomic analysis, with the experimental workflow summarized in Figure 1C.

### 3.2. Identification of DAPs of IPEC-J2 Cells in Response to PoRV G9P[23] Infection

**Figure 2:**
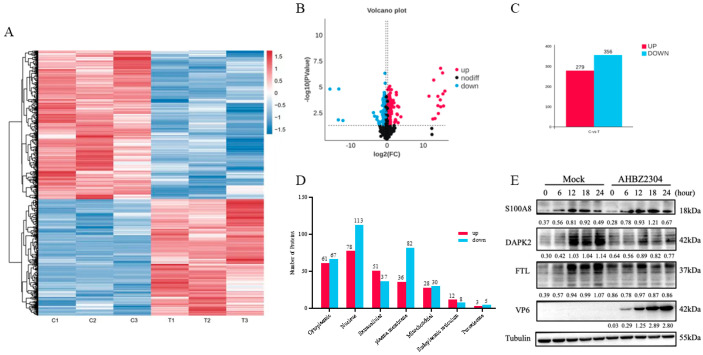
Identification and validation of proteomics data. (**A**) Hierarchical clustering analysis of DAPs. Relative expression level of DAPs is represented as color range from low level (blue) to high level (red). (**B**) Volcano plot of DAPs. Unchanged, up-abundant, and down-abundant proteins were represented by dark red and blue points, respectively. (**C**) The data statistics of DAPs with AHBZ2304 infection. (**D**) Subcellular localization prediction of DAPs in IPEC-J2 cells during AHBZ2304 infection. (**E**) Confirmation of DAPs by Western blot analysis. Western blot analysis of the SA100A8, DAPK2, and FTL protein expression levels in AHBZ2304- or mock-infected IPEC-J2 cells at 6 HPI, 12 HPI, 18 HPI, and 24 HPI (MOI = 1). Tubulin was used as a loading control. The intensity ratio between the corresponding bands (PoRV-infected/mock band) was measured by densitometric scanning and normalized to the intensity of the Tubulin bands in each experiment.

Through the 4D DIA-based quantitative proteomic analysis, a total of 152,419 peptides and 8725 cellular proteins were identified through mapping against the Sus scrofa (large white) reference genome (Ensemble database) from mock-infected and PoRV-infected IPEC-J2 cells at 24 HPI. Comparative analysis revealed 635 DAPs based on the fold change and *p*-value (<0.05), comprising 279 more abundant proteins (>1.2) and 356 under-detected proteins (<0.83). These results were visualized by clustering the samples according to differential group and by constructing a volcano plot of the DAPs (Figure 2A–C). Among the DAPs, XP_005661217.1 was the most up-abundant with log2 (fold change) > 16.0, while XP_020947047.1 was the most down-abundant with log2 (fold change) < −15.0 (Table 1). All the DAPs are listed in Supplementary Table S2.

### 3.3. Subcellular Localization and Validation of the DAPs by Western Blot

To elucidate the functional implications of differentially abundant proteins (DAPs), subcellular localization predictions were performed for DAPs identified in PoRV-infected IPEC-J2 cells. As shown in Figure 2D, the DAPs were categorized into seven subcellular compartments. Specifically, 61 up-abundant proteins and 67 down-abundant proteins were localized to the cytoplasm; 78 up-abundant proteins and 113 down-abundant proteins to the nucleus; 51 up-abundant proteins and 37 down-abundant proteins to extracellular; 36 up-abundant proteins and 82 down-abundant proteins to the plasma membrane; 28 up-abundant proteins and 30 down-abundant proteins to the mitochondria; 12 up-abundant proteins and 8 down-abundant proteins to the endoplasmic reticulum (ER); and 3 up-abundant proteins and 5 down-abundant proteins to the peroxisome. To validate the proteomic findings, a Western blot analysis was performed for three DAPs: pro-inflammatory mediator S100A8, apoptosis-associated DAPK2, and iron metabolism regulator FTL. Consistent with proteomic data, PoRV infection significantly increased S100A8 expression while reducing DAPK2 and FTL levels compared to mock-infected cells (Figure 2E and Supplementary Figure S1).

### 3.4. GO and KEGG Analysis of the DAPs

**Figure 3:**
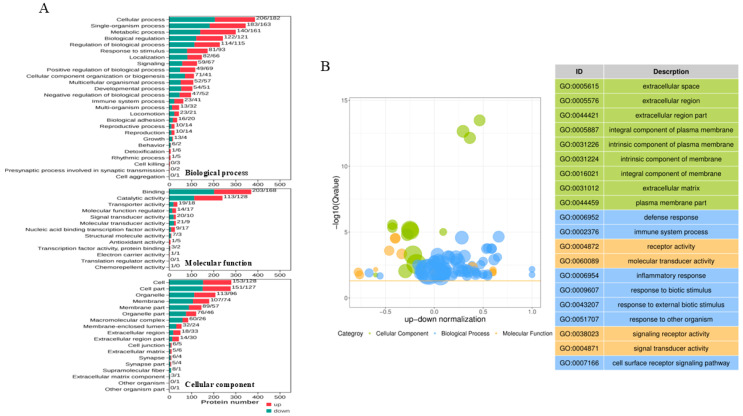
Gene Ontology (GO) annotation analysis of DAPs identified in IPEC-J2 cells with AHBZ2304 infection. (**A**) The proteins were annotated into biological process (BP), cellular component (CC), and molecular function (MF). The ordinate text indicates the name and classification of GO terms. The red and green columns represent the up-abundant and down-abundant proteins, respectively, with the number of altered proteins being marked on top of each column. (**B**) The bubble plots of Gene Ontology and top 20 of GO term.

The DAPs involved in the biological process (BP), molecular function (MF), and cellular component (CC) were analyzed according to the Gene Ontology (GO) annotation. As shown in Figure 3A, the biological process annotation analysis revealed that the DAPs were mainly associated with the cellular process, metabolic process, biological process and regulation, and response to stimulus. Notably, 41 up-abundant proteins and 23 downabundant proteins were involved in the immune system process. Within the MF category, most of the DAPs were enriched in the binding function and catalytic activity. For the CC category, the proteins were predicted to be mainly distributed within 10 cellular components, including the cell, cell part, organelle, membrane, membrane part, and so on. A top 20 GO term analysis highlighted immune-related processes as most significantly enriched, including defense response, immune system process, and inflammatory response. In terms of CCs, the “extracellular space”, “extracellular region”, and “extracellular space part” were most associated with PoRV infection. Among MF terms, the DAPs were enriched in receptor activity, molecular transducer activity, signaling receptor activity, and signaling transducer activity (Figure 3B).

**Figure 4:**
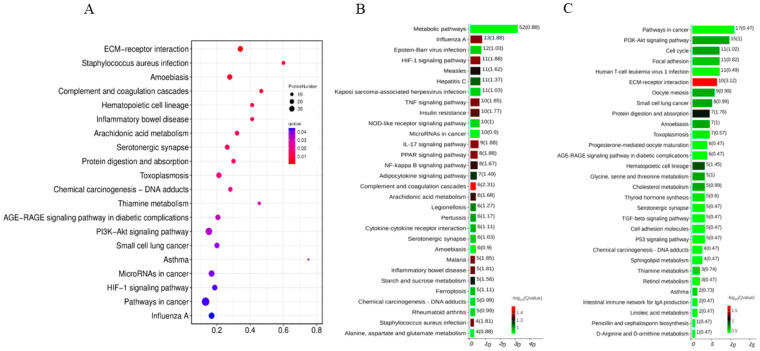
Kyoto Encyclopedia of Genes and Genomes (KEGG) annotation analysis of DAPs identified in IPEC-J2 cells with AHBZ2304 infection. (**A**) The statistics of the top 20 KEGG pathways of DAPs. The size of each dot represents the number of proteins enriched in the corresponding pathway. The color of each dot corresponds to the value which indicates the significant level of change in each pathway. (**B**) The top 30 significant pathways of the significantly up-abundant proteins. (**C**) The top 30 significant pathways of the significantly down-abundant proteins, with the percentage of altered proteins and change value being marked on top of each column.

In addition, KEGG pathway analyses were performed to explore the underlying signaling pathways among the DAPs. All the DAPs could be classified into six clusters, including metabolism, genetic information processing, environmental information processing, cellular processes, organismal systems, and human diseases (Supplementary Figure S2). Notably, a KEGG pathway enrichment analysis revealed high correlations between PoRV infection and the ECM–receptor interaction, complement and coagulation cascades, inflammatory bowel disease, arachidonic acid metabolism, and the PI3K-Akt signaling pathway (Figure 4A). For the up-abundant proteins, the top 30 relevant pathways were shown in Figure 4B, and the metabolic pathway, HIF-1 signaling pathway, TNF signaling pathway, IL-17 signaling pathway, etc., are worth being further studied. For the down-abundant proteins, the signaling pathways of interest included the PI3K-Akt signaling pathway, cell cycle, cholesterol metabolism, TGF-β signaling pathway, p53 signaling pathway, and so on (Figure 4C).

### 3.5. Inflammatory Cytokine Expression in Response to PoRV G9P[23] Infection

**Figure 5:**
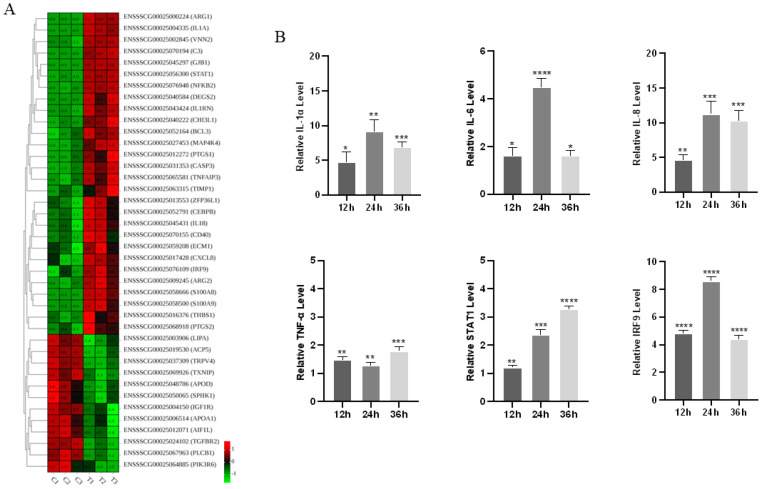
Identification and validation of inflammatory-associated proteins. (**A**) Hierarchical clustering analysis of DAPs associated with inflammatory proteins. The expression level of DAPs is represented as red and green color, respectively. (**B**) Quantitative real-time PCR (qPCR) analysis of the relative mRNA expression level of IL-1α, IL-6, IL-8, TNF-α, STAT1, and IRF9 in AHBZ2304- or mock-infected IPEC-J2 cells at 12 HPI, 24 HPI, and 36 HPI (MOI = 1). GAPDH was used as reference genes. Data are shown as mean (SD) with *n* = 3 per group. * *p* < 0.05, ** *p* < 0.01, *** *p* < 0.001, and **** *p* < 0.0001 were considered statistically significant.

As a representative inflammatory bowel disease, the PoRV G9P[23] infection induced significant dysregulation of inflammatory mediators, with numerous up-abundant and down-abundant proteins linked to inflammatory pathways identified among the 635 DAPs (Figure 5A). To further validate the obtained proteomic data, mRNA levels of several inflammation-associated genes were quantified by qPCR in mock-infected and PoRV-infected IPEC-J2 cells (MOI = 1). Total cellular RNA was collected and extracted at 12, 24, and 36 h post-infection. As shown in Figure 5B, consistent with the proteomic results, transcriptional levels of IL-1α, IL-6, IL-8, TNF-α, STAT1, and IRF9 were significantly up-abundant (*p* < 0.05) at all time points. Taken together, these results corroborate the reliability of the quantitative proteomic dataset and underscore the pivotal role of inflammatory signaling in PoRV pathogenesis.

## 4. Discussion

Porcine rotavirus (PoRV) has re-emerged as a significant swine pathogen in China with a marked increase in clinical cases and economic losses in recent years [5,7,11]. The G9P[23] strain AHBZ2304, previously isolated and characterized in our group, exhibited high pathogenicity in neonatal piglets [11], yet the molecular mechanisms underlining its virulence and host interactions remain poorly understood. Proteomic approaches have been widely employed to dissect virus–host interplay, providing critical insights into pathogenesis and facilitating the development of targeted antiviral strategies. Recently, Tao et al. performed a lipidomic analysis of PoRV-infected IPEC-J2 cells, revealing a significant upregulation of ceramides, which possess potent antiviral activity [20]. Therefore, we demonstrated that IPEC-J2 cells derived from the jejunum of newborn piglets could be successfully infected with AHBZ2304 and then used the cell line as an in vitro model for investigating PoRV–host interactions in the present study.

Here, the 4D-DIA quantitative proteomics approach was applied to identify the proteomic alterations in IPEC-J2 cells at 24 h post-PoRV infection. A total of 8725 cellular proteins were identified, with 635 proteins showing differential abundance (fold change > 1.2 or <0.83 and *p*-value < 0.05). To ensure the reliability of the DAPs data, one up-abundant (S100A8) and two down-abundant proteins (DAPK2 and FTL) were selected for Western blot validation. Death-associated kinase 2 (DAPK2), a member of the DAPK family, plays essential roles in various cellular processes, including apoptosis and autophagy [21]. The downregulation of DAPK2 suggested that PoRV may subvert host cell death pathways to sustain replication. Ferritin light chain (FTL), a component of ferritin involved in maintaining iron homeostasis [22], was also down-abundant, indicating a aberrant iron metabolism and the activation of ferroptosis following PoRV infection. S100A8, a Ca^2+^-binding protein from the S100 family, has recently garnered attention for its role in mitochondrial dysfunction, apoptosis, and inflammatory response [23]. The upregulation of S100A8 was indicative of early-stage biological dysfunction in response to PoRV infection. These three DAPs were validated, confirming the reliability of our proteomics data. Furthermore, several proteins exhibiting large fold changes attracted our attention. For example, proline-rich 15 (PRR15), a crucial gene involved in gastrointestinal neoplasia and colon cancer [24], was markedly induced (over 15-fold), while the tumor necrosis factor interacting protein (TRAIP), a replisome-associated E3 ubiquitin ligase crucial for maintaining genome integrity, was significantly down-abundant. Other proteins associated with apoptosis, autophagy, endoplasmic reticulum (ER) stress, and mitochondrial function were also identified (Supplementary Table S2). These results of DAPs highlighted the complexity of PoRV–host interactions and warrant focused investigation into mechanisms driving pathogenesis.

Innate immune responses, particularly interferon production and the secretion of inflammatory cytokines, represent the first line to combat the invading virus. Our results showed that the defense response, immune system process, and inflammatory response were markedly enriched by GO-enrichment analyses (Figure 3B). Notably, the significant upregulation of interleukin-1A (IL-1A) and interferon-induced protein with tetratricopeptide repeats 2 (IFIT2) indicated the activation of innate immune responses in PoRV-infected IPEC-J2 cells (Table 1). PoRV is a well-known pathogen of inflammatory bowel disease, and our previous study suggested that the overexpression of inflammatory cytokines contribute to PoRV pathogenicity, so inflammatory responses were emphasized here. As illustrated by the heatmap of Figure 5A, various inflammatory-associated proteins (IL-1α, IL-8, STAT1, PTGS1/2, TRPV4, TXNIP, etc.) were up-abundant or down-abundant following PoRV infection, participating in multiple signaling pathways such as the HIF-1 signaling pathway, TNF signaling pathway, IL-17 signaling pathway, and NF-κB signaling pathway (Figure 4B). Previous studies by Wu et al. demonstrated that the PEDV M or E protein could induce the upregulation of IL-8 and trigger the activation of NF-κB [25], while Guo et al. further demonstrated that IL-8 expression enhances cytosolic Ca^2+^ levels in epithelial cells, facilitating PEDV replication through internalization and egress process [26]. Additionally, Berberine, Lactobacillus acidophilus, and Montmorillonite Powder have been reported to inhibit rotavirus replication and alleviate rotavirus-induced gastroenteritis through anti-inflammatory mechanisms [27,28]. Thus, the abnormal inflammatory responses mediated by PoRV infection and their underlining mechanisms warrant further investigation. Recently, a growing body of evidence suggests that ferroptosis is involved in inflammatory responses [29]. Banerjee et al. revealed that PoRV infection triggers ferroptotic cell death via the SLC7A11-AS1/xCT axis to facilitate viral propagation [30], while Zhao et al. showed that the 1,25D3 treatment significantly eliminates RV-induced ferroptosis [31]. Interestingly, in addition to FTL, various other ferroptosis-associated proteins, such as ACSL4, DMT1, MMP7, PARP14, ACOD1, SLC38A5, and TXNIP, were identified among the DAPs (Supplementary Table S2). The regulatory mechanisms governing ferroptosis and inflammation during PoRV infection represent a promising area of future research [32].

It is noteworthy that porcine rotaviruses may have evolved multiple strategies to antagonize the host’s innate immune response. Interleukin-1 receptor associated kinase 1 (IRAK1) is essential for sensing pathogen-associated molecular patterns (PAMPs). In alphavirus–host interactions, the capsid-IRAK1 interaction is conserved, enabling alphaviruses to evade innate immune detection and activation prior to viral gene expression [33]. Yu et al. revealed that the Newcastle disease virus (NDV) reduced coiled-coil-helix-coiled-coil-helix domain containing 10 (CHCHD10) expression to impede mitochondrial fusion and suppress IFN-β production [34]. Tripartite motif-containing E3 ubiquitin ligase TRIM4 and TRIM41 are required for innate antiviral response. Zhang et al. revealed that the SARS-CoV-2 nonstructural protein 8 (nsp8) acts as a suppressor of antiviral innate immune and inflammatory responses to promote the infection of SARS-CoV-2 by impairing TRIM4-mediated K63-linked polyubiquitination [35]. Patil et al. reported that TRIM41 is a constitutively expressed intrinsic influenza A virus (IAV) restriction factor that targets NP for ubiquitination and protein [36]. In the present study, IRAK1, CHCHD10, TRIM4, and TRIM41 were significantly aberrant in PoRV-infected IPEC-J2 cells, and the biological functions hidden behind the changes merit further exploration.

## 5. Conclusions

Taken together, the present study systematically analyzed the global protein profiles of PoRV-infected IPEC-J2 cells using a 4D-DIA proteomic approach. A total of 635 DAPs, comprising 279 more abundant and 356 under-detected proteins, were identified at 24 h post-infection with the porcine rotavirus strain AHBZ2304 (G9P[23]). Bioinformatics analyses further revealed the involvement of several crucial cellular processes and signaling pathways in PoRV infection, highlighting the need for further in-depth investigations of the identified proteins and pathways. Our current data and findings provide valuable insights into the pathogenic and defense mechanisms underlying the interaction between porcine rotavirus and the host.

## Figures and Tables

**Table 1:** The top 10 differentially abundant proteins in AHBZ2304-infected IPEC-J2 cells.

Accession	Log_2_(FC)	*p*-Value	Description
Up-abundant			
XP_005661217.1	16.00	0.00002	L-amino-acid oxidase-like
XP_003134861.1	15.64	0.00067	proline-rich protein 15
NM_214029.1	15.47	0.00005	Sus scrofa interleukin 1 alpha
XP_003357445.1	15.37	0.00000	solute carrier family 45 member 3
NP_001302587.1	14.86	0.00082	interferon-induced protein with tetratricopeptide repeats 2
XP_003135210.1	14.82	0.00000	gap junction beta-1 protein
XP_025256764.1	14.74	0.00017	lysozyme C isoform X2
XP_020955360.1	14.21	0.00359	C4-monooxygenase DES2 isoform X2
XP_020922632.1	14.08	0.00062	membrane primary amine oxidase
XP_020923513.1	14.08	0.00008	arachidonate 15-lipoxygenase B
Down-abundant			
XP_020947047.1	−15.67	0.00002	structural maintenance of chromosomes protein 1B isoform X1
XP_020937290.1	−13.36	0.01461	protein amnionless isoform X1
XP_020933783.1	−13.23	0.00002	uncharacterized protein
XP_020945789.1	−12.09	0.01691	dual specificity protein phosphatase CDC14A isoform X2
XP_014683568.1	−3.68	0.00297	BTB/POZ domain-containing protein 19 isoform X5
XP_005670995.1	−3.37	0.00642	cell division control protein 45 homolog isoform X3
XP_020940636.1	−2.73	0.01321	probable threonine tRNA ligase 2
XP_020948973.1	−2.69	0.00755	death-associated protein kinase 2 isoform X1
XP_013837163.1	−2.23	0.02169	E3 ubiquitin-protein ligase TRAIP isoform X1
XP_005662396.1	−2.22	0.02167	sulfotransferase 1C4

## Data Availability

The original contributions presented in the study are included in the article/Supplementary Materials, and the proteomics data obtained in this study were deposited to the iProx database with the number PXD063141 (https://www.iprox.cn/page/project.html?id=IPX0011590000 (accessed on 21 April 2025)).

## Supplementary Materials

The following supporting information can be downloaded at: https://www.mdpi.com/article/10.3390/vetsci12100946/s1.
