# Supplementary figures and images for: 4D-DIA Proteomic Analysis of IPEC-J2 Cells Infected with Porcine Group A Rotavirus G9P[23] Strain
Source: Vet Sci. 2025 Sep 30;12(10):946. doi: 10.3390/vetsci12100946 (PMC12567895; doi:10.3390/vetsci12100946)

## Slide 1
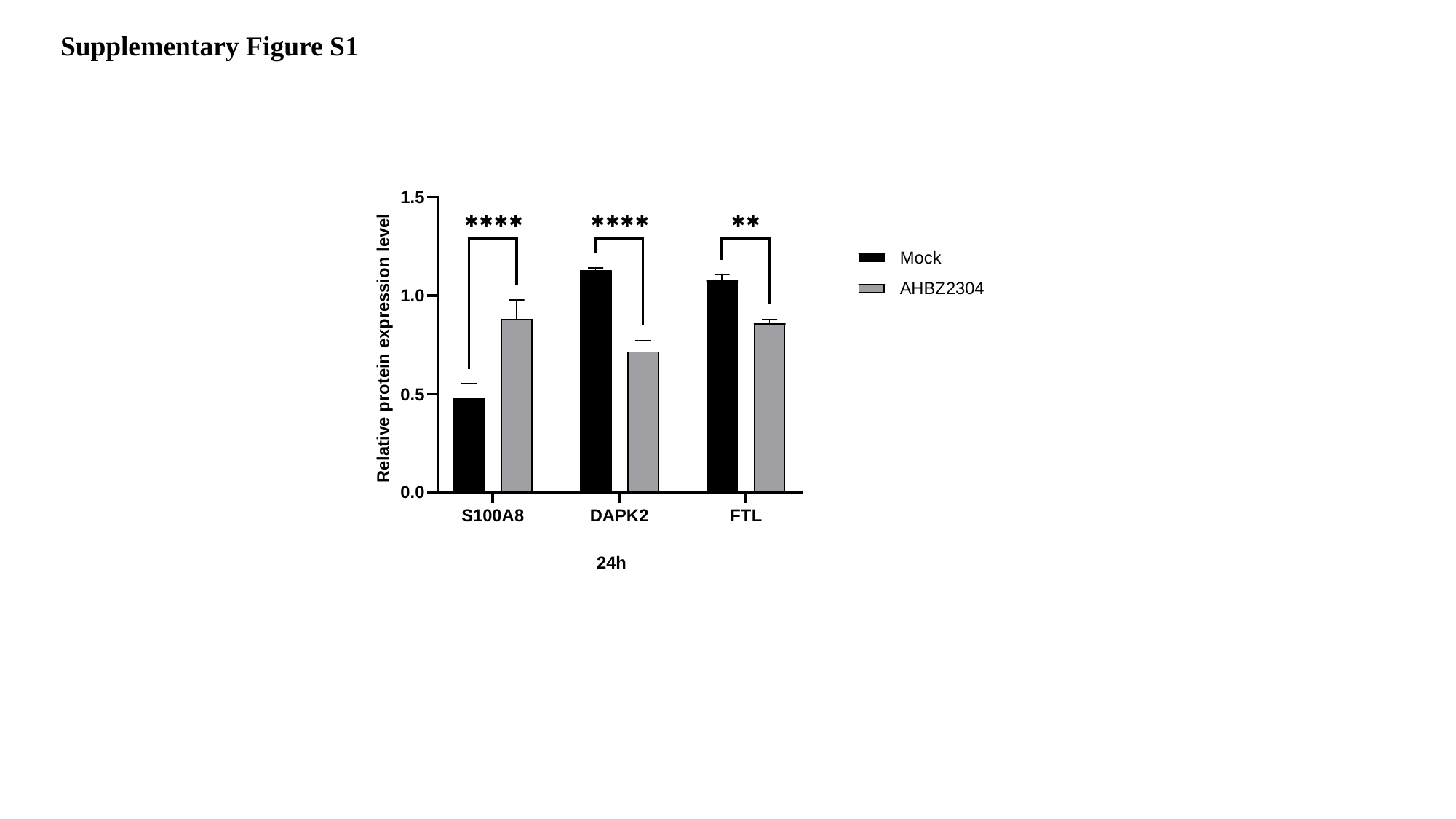

Supplementary Figure S1

## Slide 2
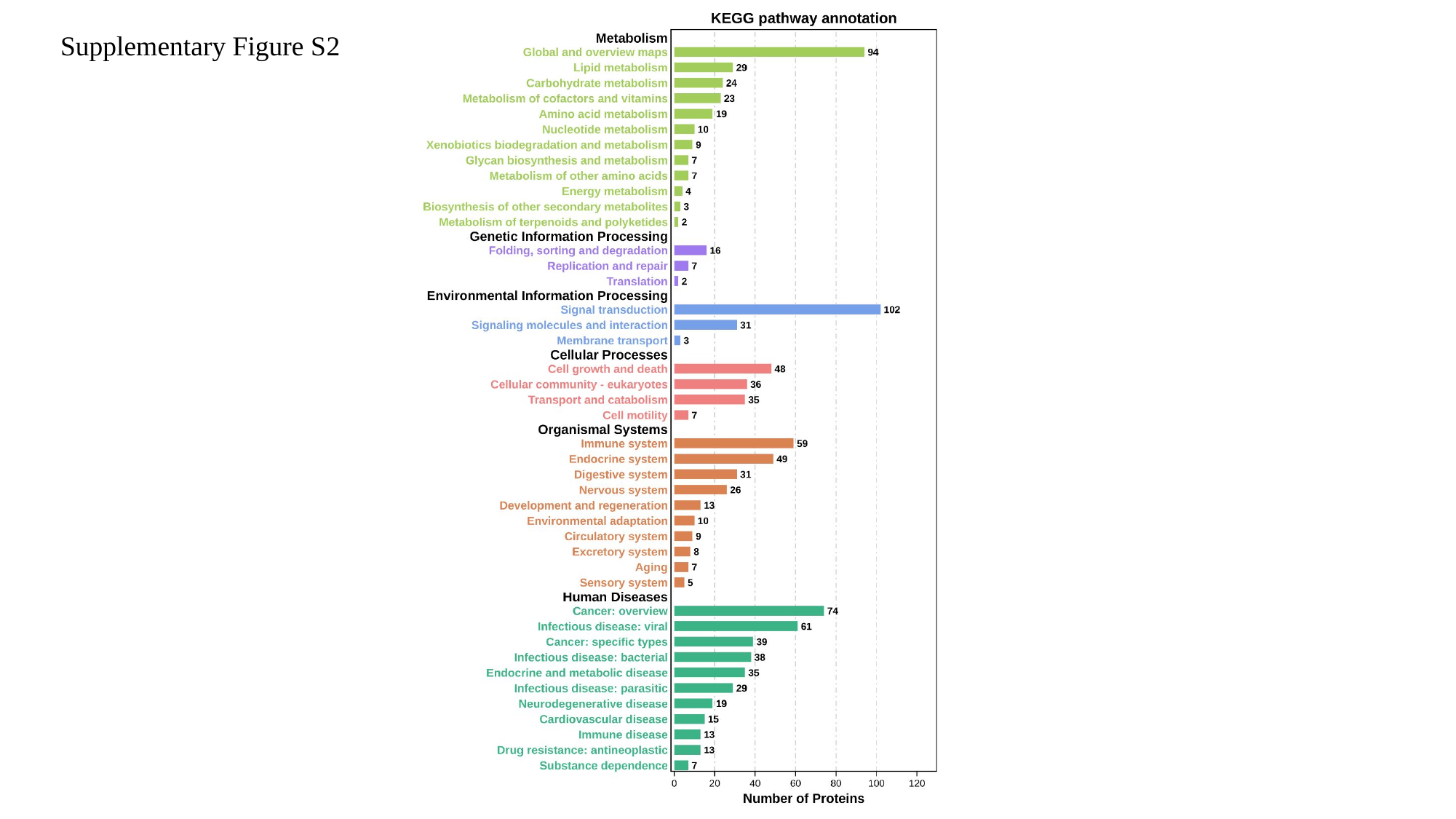

Supplementary Figure S2

Supplement: Supplementary file 1 [file vetsci-12-00946-s001.zip › vetsci-3851621-supplementary1.pptx]
